# Mathematical Modelling of HIV-HCV Coinfection Dynamics in Absence of Therapy

**DOI:** 10.1155/2020/2106570

**Published:** 2020-10-06

**Authors:** Edison Mayanja, Livingstone S. Luboobi, Juma Kasozi, Rebecca N. Nsubuga

**Affiliations:** ^1^Department of Mathematics, Makerere University, P.O. Box 7062, Kampala, Uganda; ^2^Institute of Mathematical Sciences (IMS), Strathmore University, P.O. Box 59857-00200, Nairobi, Kenya; ^3^Independent Researcher, Kampala, Uganda

## Abstract

Globally, it is estimated that of the 36.7 million people infected with human immunodeficiency virus (HIV), 6.3% are coinfected with hepatitis C virus (HCV). Coinfection with HIV reduces the chance of HCV spontaneous clearance. In this work, we formulated and analysed a deterministic model to study the HIV and HCV coinfection dynamics in absence of therapy. Due to chronic stage of HCV infection being long, asymptomatic, and infectious, our model formulation was based on the splitting of the chronic stage into the following: before onset of cirrhosis and its complications and after onset of cirrhosis. We computed the basic reproduction numbers using the next generation matrix method. We performed numerical simulations to support the analytical results. We carried out sensitivity analysis to determine the relative importance of the different parameters influencing the HIV-HCV coinfection dynamics. The findings reveal that, in the long run, there is a substantial number of individuals coinfected with HIV and latent HCV. Therefore, HIV and latently HCV-infected individuals need to seek early treatment so as to slow down the progression of HIV to AIDS and latent HCV to advanced HCV.

## 1. Introduction

Human immunodeficiency virus (HIV) is a virus that weakens the immune system by attacking the CD4^+^ T-cells. Once HIV destroys these cells, it becomes harder for the body to fight off other infections [1]. Not only does HIV attack CD4^+^ T-cells, but it also uses these cells to multiply the virus. Hepatitis C infection is a liver disease caused by hepatitis C virus (HCV) [2]. HCV and HIV are both blood borne viruses, acquired through exposure to HCV and HIV-infected blood, respectively.

Despite the availability of antiretroviral therapy (ART), HIV-infected individuals may not be on ART because they may not be diagnosed or if diagnosed they may choose to delay ART initiation. Additionally, in low-income countries, some HIV-infected individuals may have no access to ART whereas others may drop out [3]. Similarly, HCV-infected individuals in the chronic stage may be undiagnosed, and thus cannot seek treatment. Indeed, screening, diagnosis, and treatment of HCV-infected individuals have been and remain a global challenge [4]. For these reasons, in this work we investigate the HIV-HCV coinfection dynamics in absence of therapy.

HIV and HCV have similar transmission routes such as the following : sharing injection drugs and needles, having unprotected sex, mother to child transmission during pregnancy or birth, blood and blood product transfusion, organ transplants from infected donors, and exposure to blood by health care professionals [5]. However, sexual transmission of HCV is debatable; whereas it is believed that HCV can be transmitted sexually, the risk is considered relatively low [2, 5–9]. On the other hand, the risk of HCV sexual transmission is increased in the case of having multiple sexual partners; sex with high-risk individuals such as prostitutes, intravenous drug users (IDUs), and men who have sex with men (MSM); HIV or a history of a sexually transmitted disease; sex during menstruation; and sexual activities which increase the risk of blood-to-blood contact like rough vaginal or anal sex [8–11]. In sub-Saharan Africa, unlike for HIV transmission where more than 90% of the transmissions are through sexual transmission [12], the principal modes of HCV transmission are unclear [6]. Globally, most recent HCV infections are in high-risk groups such as MSM [7]. Thus, in this work we only considered transmission of HIV and HCV through sexual acts among sexually active individuals.

HCV infection is often described as acute or chronic [2]. It is estimated that about 20% to 30% of people infected with acute HCV can clear the virus spontaneously [13], whereas 85% become chronic carriers [2, 14, 15]. It is estimated that 3-4 million people are infected with HCV every year [16]. In 2015, it was estimated that about 71 million people were living with chronic HCV whereas approximately 399,000 people died from hepatitis C globally [17]; in Uganda, 2.7% have HCV [7]. Further in 2015, globally, it was estimated that of the 36.7 million people that were HIV positive, 6.3% had been coinfected with HCV [17]. Coinfection with HIV reduces the chance of spontaneous clearance of HCV [18].

Over the years, mathematical models have greatly been used to understand the dynamics of infectious diseases within an individual or groups of individuals and to suggest intervention strategies. Several scholars have developed mathematical models for coinfection of various diseases to determine the impact of a given disease on the natural history of the other (s) and vice versa. For example, Shah et al. [1] and Bhunu et al. [19] studied coinfection of HIV and tuberculosis; Nannyonga et al. [20] and Nyabadza et al. [21] studied coinfection of HIV and malaria; Gurmu et al. [12] and Verma et al. [22] studied coinfection of HIV and human papillomavirus (HPV); Sanga et al. [23] studied coinfection of HIV and cervical cancer; Carvalho and Pinto [5, 14], Bhunu and Mushayabasa [15], Zerehpoush and Kheiri [16], and Sanchez et al. [18] studied coinfection of HIV and HCV.

Bhunu and Mushayabasa [15] studied a mathematical model for HIV-HCV coinfection in which they aimed at investigating the possible impact of HIV on HCV and vice versa. These authors showed that HCV has an ongoing prolonged negative effect on the health of the population, irrespective of their HIV status. The authors inferred that HCV control measures should be reinforced in resource-limited settings. Carvalho and Pinto [14] developed a mathematical model for HIV-HCV coinfection that included vertical transmission for the case of HIV. These authors showed that there was a change on the dynamical behaviour of the model due to change in the values of the relevant parameters. They inferred possible measures that could be taken to reduce the number of infected individuals. Carvalho and Pinto [5] developed a mathematical model for HIV-HCV coinfection in men who have sex with men (MSM). The model included screening, awareness and unawareness of HIV infection, and effective protection against HIV and HCV by condom use. The authors showed that there was a change on the dynamical behaviour of the model due to variations in the values of the relevant parameters. They inferred that MSM were at a risk of HCV reinfection after successful treatment and clearance of HCV. These authors suggested specific measures to be considered in order to reduce HIV and HCV infections, such as distributing more condoms to individuals and encourage condom use during anal intercourse and developing campaign to sensitize individuals on the dangers of having many sexual partners.

The existing HIV-HCV coinfection mathematical models, for example, by Carvalho and Pinto [5, 14] and Bhunu and Mushayabasa [15], have been developed by either ignoring infection stages or considering HCV in two stages of infection, i.e., acute and chronic infection. However, the chronic stage of HCV infection requires reasonable attention because it is very long, and yet those infected are asymptomatic and infectious. It is this knowledge gap that we intend to address in this work. Our model formulation is based on the splitting of the chronic stage into two stages namely, before onset of cirrhosis and its complications and after onset of cirrhosis. We believe the model formulated in this work can be extended to other biological systems and disease dynamics such as studying the dynamics of hepatitis B virus (HBV) infection.

## 2. Model Formulation

In the proposed model, we split the HCV chronic stage into two categories. One category is the latent HCV characterized by undiagnosed long infectious period, and the second category is the advanced HCV characterized by onset of cirrhosis and its related complications. The proposed model is comprised of eight compartments, namely, (a) of singly disease-infected individuals: the susceptible, *S*(*t*); acutely HCV-infected, *I*_*C*_(*t*); latent HCV, *C*_*L*_(*t*); advanced HCV, *C*_*C*_(*t*); infected with HIV but without AIDS symptoms, *I*_*H*_(*t*); and those with full-blown AIDS symptoms, $\tilde{A}\left(t\right)$; (b) of coinfected individuals: coinfected with HIV and acute HCV, *I*_HC_(*t*) and those coinfected with HIV and latent HCV, *I*_HC_*L*__(*t*).

We made the following assumptions regarding the transmission of HCV and HIV: HCV or HIV transmission is through sexual acts, and hence susceptible individuals are sexually active individuals at age *a*  and above (the mean age of sexual debut in Uganda is 16 years [24]); for simplicity, we assume that HIV and HCV cannot be transmitted simultaneously; individuals that spontaneously clear acute HCV can be reinfected with acute HCV since previous infection does not confer immunity [15]; due to frailty, full-blown AIDS patients cannot get new sexual partners nor engage in sexual activities, and hence do not transmit HIV as well as advanced HCV individuals. We suppose that there is a constant recruitment rate ∧ into the susceptible class and a constant natural mortality at a rate *μ* in all classes. Susceptible individuals are infected with HIV at per capita rate *π*_*H*_ which depends on the average number of sexual partners acquired per year, $\tilde{c}$, HIV transmission probability per sexual contact (*β*_1_) and proportion infected with HIV. Similarly, susceptible individuals are infected with HCV at per capita rate *π*_*C*_, where *β*_2_ is the HCV transmission probability per sexual contact. The forces of infection associated with HIV and HCV infection are thus given by (1) and (2), respectively:

(1) $$\begin{array}{c} \pi_{H}=\frac{\tilde{c}\beta_{1}\left[I_{H}+\omega \left(I_{\text{HC}}+I_{{\text{HC}}_{L}}\right)\right]}{N}, \end{array}$$

(2) $$\begin{array}{c} \pi_{C}=\frac{\tilde{c}\beta_{2}\left[I_{C}+C_{L}+\rho \left(I_{\text{HC}}+I_{{\text{HC}}_{L}}\right)\right]}{N}, \end{array}$$

where *ω* > 1 is the enhancement factor for increased risk of being infected with HIV by a dually infected individual; parameter *ρ* > 1 is the enhancement factor for increased risk of being infected with HCV by an individual coinfected with HIV and HCV. Individuals coinfected with HIV and HCV have higher viral loads of HIV and HCV as compared to those infected with only one of the two viruses. This may increase their risk of transmission of each of the viruses [25]. Both *ω* and *ρ* model the fact that coinfected individuals are more infectious than their counterparts who are singly infected [15].

Susceptible individuals, once infected with HIV, enter the HIV only infected class, *I*_*H*_(*t*). Individuals in the class *I*_*H*_(*t*) progress to AIDS class at a rate *α*. Individuals in the AIDS class not only die from natural death, but also from AIDS-induced deaths at a rate $\sigma_{\tilde{A}}$. On the other hand, susceptible individuals once infected with HCV enter the class of acute HCV-infected individuals, *I*_*C*_(*t*). Some of the Individuals in  *I*_*C*_(*t*) class clear the acute HCV spontaneously at a rate *τ* while the others progress to latent HCV class, *C*_*L*_(*t*), at a rate *γ*. Then, individuals from *C*_*L*_(*t*) enter the class of individuals in advanced HCV, *C*_*C*_(*t*), at a rate *ϕ*. Individuals in advanced HCV class die from natural death and from advanced HCV at a rate *σ*_*C*_*C*__.

The presence of HIV may increase the risk of acquiring HCV; thus, individuals living with HIV are at higher risk of contracting HCV than those without HIV because HIV weakens the immune system, which leaves the body more vulnerable to other infections and illnesses [26]. Furthermore, since HIV and HCV are transmitted in similar ways, individuals who are infected with HIV are at a high risk of exposure to HCV and vice versa. Therefore, amplification parameters, *k*_*i*=1,2,3_ > 1, have been included to cater for the increased risk of getting infected with HCV for those individuals who are already infected with HIV and vice versa [15] as described in the detail below.

When individuals in classes *I*_*C*_(*t*) and *I*_*H*_(*t*) engage in sexual contact, they are likely to become dual infected with both HIV and acute HCV, where individuals who are infected with HIV only and are not yet in the AIDS class of disease progression, become infected with acute HCV at a rate *k*_2_*π*_*C*_ and enter the class of those individuals coinfected with HIV and acute HCV, *I*_HC_(*t*), whereas those who are infected with acute HCV become coinfected with HIV at a rate *k*_1_*π*_*H*_. An amplification parameter *k*_1_ > 1 has been introduced to cater for the increased risk of getting infected with HIV for individuals who are already infected with acute HCV. On the other hand, an amplification parameter *k*_2_ > 1 has been introduced to cater for the increased risk of getting infected with acute HCV for individuals who are already infected with HIV. In addition, some of the individuals who are coinfected with acute HCV and HIV can spontaneously clear acute HCV at a rate *rτ* and return back to the *I*_*H*_(*t*) class. Due to the fact that the probability of spontaneous clearance of the HCV virus is reduced in the case of coinfection [18], a reduction parameter *r* < 1 has been introduced to cater for the reduced risk of spontaneous clearance of acute HCV due to the coinfection of acute HCV and HIV.

When individuals in classes *C*_*L*_(*t*) and *I*_*H*_(*t*) engage in a sexual encounter, individuals in class  *C*_*L*_(*t*) are projected to become coinfected with HIV at a rate *k*_3_*π*_*H*_ to enter the class of individuals who are dually infected with HIV and latent HCV, *I*_HC_*L*__(*t*). An amplification parameter *k*_3_ > 1 has been introduced to account for the increased risk of getting infected with HIV for individuals who are infected with latent HCV, whereas individuals who are in the class *I*_*H*_(*t*) get infected with acute HCV at a rate *k*_2_*π*_*C*_ to enter class *I*_HC_(*t*). Individuals who are coinfected with HIV and acute HCV and fail to spontaneously clear the acute HCV progress to the HIV-latent HCV-coinfected class at a rate *θ*.

The parameters presented in the description of HIV-HCV coinfection dynamics are summarised in Table 1.

**Figure 1:**
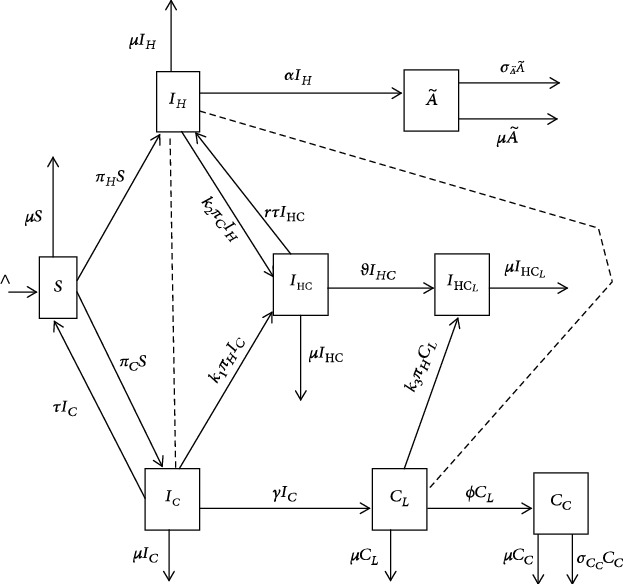
Flow diagram for HIV-HCV coinfection dynamics. Solid arrows indicate movement from one compartment to another, and dashed connections show the interaction between the connected compartments.

The HIV-HCV coinfection dynamics are presented as in the compartment flow diagram in Figure 1.

From the compartmental diagram in Figure 1, the associated mathematical model is as in Equations (3)–(10).

(3) $$\begin{array}{c} \frac{dS}{dt}=\wedge +\tau I_{C}-\pi_{C}S-\pi_{H}S-\mu S, \end{array}$$

(4) $$\begin{array}{c} \frac{dI_{H}}{dt}=\pi_{H}S+r\tau I_{\text{HC}}-k_{2}\pi_{C}I_{H}-\alpha I_{H}-\mu I_{H}, \end{array}$$

(5) $$\begin{array}{c} \frac{dI_{C}}{dt}=\pi_{C}S-k_{1}\pi_{H}I_{C}-\gamma I_{C}-\tau I_{C}-\mu I_{C}, \end{array}$$

(6) $$\begin{array}{c} \frac{dI_{\text{HC}}}{dt}=k_{2}\pi_{C}I_{H}+k_{1}\pi_{H}I_{C}-\mu I_{\text{HC}}-r\tau I_{\text{HC}}-\theta I_{\text{HC}}, \end{array}$$

(7) $$\begin{array}{c} \frac{dI_{{\text{HC}}_{L}}}{dt}=\theta I_{\text{HC}}+k_{3}\pi_{H}C_{L}-\mu I_{{\text{HC}}_{L}}, \end{array}$$

(8) $$\begin{array}{c} \frac{dC_{L}}{dt}=\gamma I_{C}-k_{3}\pi_{H}C_{L}-\mu C_{L}-\emptyset C_{L}, \end{array}$$

(9) $$\begin{array}{c} \frac{d\tilde{A}}{dt}=\alpha I_{H}-\sigma_{\tilde{A}}\tilde{A}-\mu \tilde{A}, \end{array}$$

(10) $$\begin{array}{c} \frac{dC_{C}}{dt}=\emptyset C_{L}-\mu C_{C}-\sigma_{C_{C}}C_{C}, \end{array}$$

where *π*_*H*_ and *π*_*C*_ are as defined in (1) and (2), respectively. The initial values of the variables of the system are as follows: *S*(0) > 0, *I*_*H*_(0) ≥ 0, *I*_*C*_(0) ≥ 0, *I*_HC_(0) ≥ 0, *I*_HC_*L*__(0) ≥ 0, *C*_*L*_(0) ≥ 0, $\tilde{A}\left(0\right)\geq 0$, and *C*_*C*_(0) ≥ 0. As indicated earlier, we assumed that AIDS cases, $\tilde{A}\left(t\right)$, are too weak, have full-blown observable symptoms, and can no longer get new sexual partners. They do not engage in sexual activity, similarly for individuals in the advanced HCV class, *C*_*C*_(*t*). Thus, Equations (3), (4), (5), (6), (7), and (8) are independent of AIDS cases, $\tilde{A}\left(t\right)$, and of the advanced HCV-infected individuals, *C*_*C*_(*t*). Therefore, compartments $\tilde{A}$ and *C*_*C*_ do not feed into any other compartments as such we excluded them from the active population. Hence, the total active population at time *t*, *N*(*t*), is given by

(11) $$\begin{array}{c} N\left(t\right)=S\left(t\right)+I_{H}\left(t\right)+I_{C}\left(t\right)+C_{L}\left(t\right)+I_{\text{HC}}\left(t\right)+I_{{\text{HC}}_{L}}\left(t\right). \end{array}$$

## 3. Model Analysis and Results

### 3.1. Basic Properties of the Model

In this subsection, we study the basic properties of the solutions of the model in Equations (3), (4), (5), (6), (7), and (8).

**Theorem 1 . (positivity of solutions).**

The solutions  *S*(*t*), *I*_*H*_(*t*), *I*_*C*_(*t*), *I*_HC_(*t*), *I*_HC_*L*__(*t*), and *C*_*L*_(*t*) of the system are nonnegative for *t* ≥ 0.

**Proof**

Let the initial values of the variables of the system of Equations (3), (4), (5), (6), (7), and (8) be nonnegative. We prove that the solution component of *S*(*t*)  is positive. Assume that there exists a first time *t*_1_ : *S*(*t*_1_) = 0, *S*′(*t*_1_) < 0 and  *S*(*t*) > 0, *I*_*H*_(*t*) > 0, *I*_*C*_(*t*) > 0, *I*_HC_(*t*) > 0, *I*_HC_*L*__(*t*) > 0, *C*_*L*_(*t*) > 0 for 0 < *t* < *t*_1_.

From (3) of the system, we have

(12) $$\begin{array}{c} \frac{dS\left(t_{1}\right)}{dt}=\wedge +\tau I_{C}\left(t_{1}\right)>0, \end{array}$$

which is a contradiction and consequently, *S*(*t*) remains positive. The others are proved in the same way.

Therefore, the solutions of the system are nonnegative whenever *t* ≥ 0.

**Theorem 2 . (invariant region).**

The region *Ω* = {(*S*(*t*), *I*_*H*_(*t*), *I*_*C*_(*t*), *I*_HC_(*t*), *I*_HC_*L*__(*t*), *C*_*L*_(*t*)) ∈ *R*_+_^6^ : *N*(*t*) ≤ max{*N*_0_, ∧/*μ*}} is positively invariant and attracting with respect to the model.

**Proof**

Let (*S*(*t*), *I*_*H*_(*t*), *I*_*C*_(*t*), *I*_HC_(*t*), *I*_HC_*L*__(*t*), *C*_*L*_(*t*)) ∈ *R*_+_^6^ be any solution of the system with nonnegative initial condition given by (*S*(0), *I*_*H*_(0), *I*_*C*_(0), *I*_HC_(0), *I*_HC_*L*__(0), *C*_*L*_(0)). The total active population is given by  *N*(*t*). Adding Equations (3), (4), (5), (6), (7), (8), we obtain

(13) $$\begin{array}{c} \frac{dN\left(t\right)}{dt}=\wedge -\mu N\left(t\right)-\alpha \;I_{H}\left(t\right)-\emptyset C_{L}\left(t\right). \end{array}$$

For *I*_*H*_(*t*) ≥ 0 and *C*_*L*_(*t*) ≥ 0 for *t* ≥ 0, we have

(14) $$\begin{array}{c} \frac{dN\left(t\right)}{dt}+\mu N\left(t\right)\leq \wedge , \end{array}$$

and

(15) $$\begin{array}{c} N\left(t\right)\leq \frac{\wedge }{\mu }+\left(N_{0}-\frac{\wedge }{\mu }\right)e^{-\mu t}, \end{array}$$

where *N*_0_ ≥ 0  is the initial total population size. Two scenarios arise:

*Scenario I*: If *N*_0_ > ∧/*μ*, then (15) implies *N*(*t*) ≤ *N*_0_ for all values of *t*.

*Scenario II*: If *N*_0_ < ∧/*μ*, then (15) implies *N*(*t*) ≤ ∧/*μ* for all values of *t*.

Therefore, *N*(*t*) ≤ max{*N*_0_, ∧/*μ*}. Every feasible solution of the model that starts in the region *Ω* = {(*S*(*t*), *I*_*H*_(*t*), *I*_*C*_(*t*), *I*_HC_(*t*), *I*_HC_*L*__(*t*), *C*_*L*_(*t*)) ∈ *R*_+_^6^ : *N*(*t*) ≤ max{*N*_0_, ∧/*μ*}} remains in the region for all values of  *t*. Hence, the region *Ω* is biologically feasible and positively invariant.

Therefore, the model is well posed epidemiologically and mathematically.

### 3.2. Basic Reproduction Numbers and Stability of Equilibria

The basic reproduction number is defined as the expected number of secondary infections produced by a single infected individual in a completely susceptible population. The basic reproduction number as computed using the next generation method is defined as the spectral radius of the next generation matrix [27]. In the computations of the basic reproduction numbers, we present two mono HIV and mono HCV submodels and then later the HIV-HCV coinfection model.

#### 3.2.1. The HIV-Free Equilibrium and Reproduction Number for HIV-Only Submodel

We set  *I*_*C*_(*t*) = *C*_*L*_(*t*) = *I*_HC_(*t*) = *I*_HC_*L*__(*t*) = 0 in system of Equations (3), (4), (5), (6), (7), and (8); thus,

(16) $$\begin{array}{c} \frac{dS_{\text{HIV}}}{dt}=\wedge -\pi_{H}S_{\text{HIV}}-\mu S_{\text{HIV}}, \end{array}$$

(17) $$\begin{array}{c} \frac{dI_{H}}{dt}=\pi_{H}S_{\text{HIV}}-\alpha I_{H}-\mu I_{H}, \end{array}$$

where $\pi_{H}=\tilde{c}\beta_{1}I_{H}/N_{\text{HIV}}$ and *N*_HIV_ = *S*_HIV_ + *I*_*H*_.

The HIV-free equilibrium is given by *ε*_HIV_^0^ = (*S*_HIV_^0^, *I*_*H*_^0^) = (∧/*μ*, 0).

The basic reproduction number for HIV-only submodel, *R*_HIV_, is equal to the product of HIV infection rate ($\tilde{c}\beta_{1}$) and average length of time an individual lives under both forces of HIV epidemic and natural mortality (1/*α* + *μ*); hence,

(18) $$\begin{array}{c} R_{\text{HIV}}=\frac{\tilde{c}\beta_{1}}{\left(\alpha +\mu \right)}. \end{array}$$

Therefore, interventions for reducing HIV infection should target on reducing $\;\tilde{c}$, *β*_1_, and increasing *α*. However, increasing *α* would imply fast progression to AIDS. This is not desirable. From an infected individual's perspective, we would concentrate on the effects of parameters $\tilde{c}$ and *β*_1_.

Using Theorem 2 [27], we establish that the HIV-free equilibrium, *ε*_HIV_^0^, is locally asymptotically stable if *R*_HIV_ < 1  and unstable otherwise.

#### 3.2.2. Global stability of HIV-Free Equilibrium for HIV-Only Submodel

To study the global behaviour of system of Equations (16) and (17), we use the theorem by Castillo-Chavez et al. [28]. Re-writing HIV-only system of Equations (16) and (17) in the form of Equation (3.1) of [28] and using the same notation as used in [28], we have

(19) $$\begin{array}{c} X=\left(S_{\text{HIV}}\right),Z=\left(I_{H}\right),F\left(X,0\right)=\left[\wedge -{\mu S}_{\text{HIV}}\right],\\ A=\left[\tilde{c}\beta_{1}-\left(\alpha +\mu \right)\right],\hat{G}\left(X,Z\right)=\left[\tilde{c}\beta_{1}I_{H}\left(1-\frac{S_{\text{HIV}}}{N_{\text{HIV}}}\right)\right]. \end{array}$$

Since 0 ≤ *S*_HIV_ ≤ *N*_HIV_, it is easy to see that $\hat{G}\left(X,Z\right)\geq 0$. This implies that the HIV-free equilibrium, *ε*_*HIV*_^0^, is globally asymptotically stable for *R*_HIV_ < 1.

#### 3.2.3. HIV Endemic Equilibrium

Here, we make an insight into the persistence of HIV, where the HIV endemic equilibrium is given by

(20) $$\begin{array}{c} \varepsilon_{\text{HIV}}^{*}=\left(S_{\text{HIV},}^{*}I_{H}^{*}\right)=\left(\frac{\wedge }{\left(\tilde{c}\beta_{1}-\alpha \right)},\frac{\wedge \left[\tilde{c}\beta_{1}-\left(\alpha +\mu \right)\right]}{\left(\alpha +\mu \right)\left(\tilde{c}\beta_{1}-\alpha \right)}\right). \end{array}$$

**Lemma 1 .**

The HIV endemic equilibrium, *ε*_HIV_^∗^, is locally asymptotically stable if *R*_HIV_ > 1 otherwise unstable.

**Proof**

The Jacobian matrix of system (16)-(17) evaluated at  *ε*_HIV_^∗^ is given by

(21) $$\begin{array}{c} J\left(\;\varepsilon_{\text{HIV}}^{*}\right)=\left[\begin{array}{cc} -\left(\tilde{c}\beta_{1}-\alpha \right) & -\left(\alpha +\mu \right)\\ \tilde{c}\beta_{1}-\left(\alpha +\mu \right) & 0 \end{array}\right].\\ \text{Now},\text{tr}\left(J\left(\varepsilon_{\text{HIV}}^{*}\right)\right)=-\left(\tilde{c}\beta_{1}-\alpha \right), \end{array}$$

then det(*J*(*ε*_HIV_^∗^)) > 0 when $\tilde{c}\beta_{1}>\left(\alpha +\mu \right)$, which implies that $R_{\text{HIV}}=\tilde{c}\beta_{1}/\left(\alpha +\mu \right)>1$. Since the trace of *J*(*ε*_HIV_^∗^) is negative and its determinant is positive when *R*_HIV_ > 1; thus, *ε*_HIV_^∗^ is locally asymptotically stable.

#### 3.2.4. Global Stability of the HIV Endemic Equilibrium

**Lemma 2 .**

If *R*_HIV_ > 1, then the HIV endemic equilibrium of HIV-only submodel, *ε*_HIV_^∗^, is globally asymptotically stable.

**Proof**

To prove global stability of the HIV endemic equilibrium for the system (16)-(17), we propose the following Lyapunov function

(22) $$\begin{array}{c} L=L\left(S_{\text{HIV}},I_{H}\right)=U_{1}\left(S_{\text{HIV}}-S_{\text{HIV}}^{*}-S_{\text{HIV}}^{*}\text{In}\left(\frac{S_{\text{HIV}}}{S_{\text{HIV}}^{*}}\right)\right)+U_{2}\left(I_{H}-I_{H}^{*}-I_{H}^{*}\text{In}\left(\frac{I_{H}}{I_{H}^{*}}\right)\right). \end{array}$$

The time derivative of the Lyapunouv function *L* is given by

(23) $$\begin{array}{c} \frac{dL}{dt}=U_{1}\left(1-\frac{S_{\text{HIV}}^{*}}{S_{\text{HIV}}}\right)\frac{dS_{\text{HIV}}}{dt}+U_{2}\left(1-\frac{I_{H}^{*}}{I_{H}}\right)\frac{dI_{H}}{dt}=U_{1}\left(1-\frac{S_{\text{HIV}}^{*}}{S_{\text{HIV}}}\right)\left(\wedge -\frac{\tilde{c}\beta_{1}I_{H}S_{\text{HIV}}}{N_{\text{HIV}}}-\mu S_{\text{HIV}}\right)+U_{2}\left(1-\frac{I_{H}^{*}}{I_{H}}\right)\left(\frac{\tilde{c}\beta_{1}I_{H}S_{\text{HIV}}}{N_{\text{HIV}}}-\left(\alpha +\mu \right)I_{H}\right). \end{array}$$

At the HIV endemic equilibrium, we have

(24) $$\begin{array}{c} \wedge =\frac{\tilde{c}\beta_{1}I_{H}^{*}S_{\text{HIV}}^{*}}{N_{\text{HIV}}^{*}}+\mu S_{\text{HIV}}^{*}\text{ and }\left(\alpha +\mu \right)=\frac{\tilde{c}\beta_{1}S_{\text{HIV}}^{*}}{N_{\text{HIV}}^{*}}. \end{array}$$

Substituting (24) in (23), expanding, and adopting the approach used in [29] of collecting positive terms together and negative terms together; we have

(25) $$\begin{array}{c} \frac{dL}{dt}=-M+K, \end{array}$$

where

(26) $$\begin{array}{c} M=U_{1}{\left(1-\frac{S_{\text{HIV}}^{*}}{S_{\text{HIV}}}\right)}^{2}\mu S_{\text{HIV}}+U_{1}\frac{\tilde{c}\beta_{1}I_{H}^{*}S_{\text{HIV}}^{*}}{N_{\text{HIV}}^{*}}\frac{S_{\text{HIV}}^{*}}{S_{\text{HIV}}}+U_{1}\frac{\tilde{c}\beta_{1}I_{H}S_{\text{HIV}}}{N_{\text{HIV}}}+U_{2}\frac{\tilde{c}\beta_{1}S_{\text{HIV}}I_{H}^{*}}{N_{\text{HIV}}}+U_{2}\frac{\tilde{c}\beta_{1}S_{\text{HIV}}^{*}I_{H}}{N_{\text{HIV}}} \end{array}$$

and

(27) $$\begin{array}{c} K=U_{1}\frac{\tilde{c}\beta_{1}I_{H}^{*}S_{\text{HIV}}^{*}}{N_{\text{HIV}}^{*}}+U_{1}\frac{\tilde{c}\beta_{1}I_{H}S_{\text{HIV}}^{*}}{N_{\text{HIV}}}+U_{2}\frac{\tilde{c}\beta_{1}I_{H}S_{\text{HIV}}}{N_{\text{HIV}}}+U_{2}\frac{\tilde{c}\beta_{1}I_{H}^{*}S_{\text{HIV}}^{*}}{N_{\text{HIV}}}. \end{array}$$

Hence, *dL*/*dt* ≤ 0 if *K* < *M*; and *dL*/*dt* = 0 when *S*_HIV_ = *S*_HIV_^∗^ and *I*_*H*_ = *I*_*H*_^∗^. Therefore, the largest invariant set in (*S*_HIV_^∗^, *I*_*H*_^∗^) ∈ *Ω* such that *dL*/*dt* = 0 is the singleton {*ε*_HIV_^∗^}, where *ε*_HIV_^∗^  is our HIV endemic equilibrium for the HIV-only submodel. By LaSalle's invariant principle [30], we conclude that *ε*_HIV_^∗^  is globally asymptotically stable if *K* < *M*.

#### 3.2.5. The HCV-Free Equilibrium and Reproduction Number for HCV-Only Submodel

We set  *I*_*H*_(*t*) = *I*_HC_(*t*) = *I*_HC_*L*__(*t*) = 0  in system of Equations (3), (4), (5), (6), (7), and (8); thus,

(28) $$\begin{array}{c} \frac{dS_{\text{HCV}}}{dt}=\wedge +\tau I_{C}-\pi_{C}S_{\text{HCV}}-\mu S_{\text{HCV}}, \end{array}$$

(29) $$\begin{array}{c} \frac{dI_{C}}{dt}=\pi_{C}S_{\text{HCV}}-\gamma I_{C}-\tau I_{C}-\mu I_{C}, \end{array}$$

(30) $$\begin{array}{c} \frac{dC_{L}}{dt}=\gamma I_{C}-\mu C_{L}-\emptyset C_{L}, \end{array}$$

where $\pi_{C}=\tilde{c}\beta_{2}\left[I_{C}+C_{L}\right]/N_{\text{HCV}}$ and *N*_HCV_ = *S*_HCV_ + *I*_*C*_ + *C*_*L*_.

The HCV-free equilibrium is given by *ε*_HCV_^0^ = (*S*_HCV_^0^, *I*_*C*_^0^, *C*_*L*_^0^) = (∧/*μ*, 0, 0).

**Lemma 3 .**

The basic reproduction number for HCV-only submodel

(31) $$\begin{array}{c} R_{\text{HCV}}=\frac{\tilde{c}\beta_{2}}{\left(\gamma +\tau +\mu \right)}\left(1+\frac{\gamma }{\left(\mu +\emptyset \right)}\right). \end{array}$$

**Proof**

Using the next generation matrix method of computing the basic reproduction number [27], we obtain the Jacobian matrices of new HCV infections, *F*_HCV_, and for the rate of transfer into and out of compartment *i* by all other processes, *V*_HCV_, evaluated at HCV-free equilibrium as

(32) $$\begin{array}{c} F_{\text{HCV}}=\left[\begin{array}{cc} \tilde{c}\beta_{2} & \tilde{c}\beta_{2}\\ 0 & 0 \end{array}\right],V_{\text{HCV}}=\left[\begin{array}{cc} \left(\gamma +\tau +\mu \right) & 0\\ -\gamma & \left(\mu +\emptyset \right) \end{array}\right]. \end{array}$$

The basic reproduction number of the HCV-only submodel, *R*_HCV_, is given by the spectral radius of the next generation matrix, *F*_HCV_*V*_HCV_^−1^, as

(33) $$\begin{array}{c} R_{\text{HCV}}=\frac{\tilde{c}\beta_{2}}{\left(\gamma +\tau +\mu \right)}\left(1+\frac{\gamma }{\left(\mu +\emptyset \right)}\right). \end{array}$$

Expressing (33) as

(34) $$\begin{array}{c} R_{\text{HCV}}=\frac{\tilde{c}\beta_{2}}{\left(\mu +\emptyset \right)}\frac{\left(\mu +\emptyset +\gamma \right)}{\left(\gamma +\tau +\mu \right)}=\frac{\tilde{c}\beta_{2}}{\left(\mu +\emptyset \right)}f\left(\gamma \right), \end{array}$$

in which *f*(*γ*) = (*μ*+∅+*γ*)/(*γ* + *τ* + *μ*) = *γ* + *d*/*γ* + *e*, where *d* = *μ* + ∅ and *e* = *μ* + *τ*.

(35) $$\begin{array}{c} \text{Now},\underset{\gamma \to +\infty }{\lim}f\left(\gamma \right)=1\text{ and }\underset{\gamma \to 0}{\lim}f\left(\gamma \right)=\frac{d}{e}. \end{array}$$

Two scenarios arise:

*Scenario I*: If *d* < *e* (that is, ∅<*τ*), HCV will go to extinction. This is because majority of the HCV acutely infected individuals will spontaneously clear of acute HCV, and in the long run, HCV will die out completely.

*Scenario II*: If *d* > *e* (that is, ∅>*τ*), HCV will persist. This is due to majority of acutely HCV-infected individuals failing to clear spontaneously and becoming latently infected. Without HCV treatment, such individuals have a prolonged stay in the HCV latent stage which leads to having a long time of infecting other individuals with HCV.

From (34) and (35), it is deduced that keeping other parameters constant and varying *γ* alone, *R*_HCV_ is bounded, that is

(36) $$\begin{array}{c} R_{\text{HCV}}\left(\gamma \right)\leq \frac{\tilde{c}\beta_{2}}{\left(\mu +\emptyset \right)}\max\left\{\frac{d}{e},1\right\}. \end{array}$$

Using Theorem 2 [27], we establish that the HCV-free equilibrium, *ε*_HCV_^0^, is locally asymptotically stable if *R*_HCV_ < 1  and otherwise unstable.

#### 3.2.6. Global Stability of HCV-Free Equilibrium for HCV-Only Submodel

We proceed like in Subsection 3.2.2. Rewriting HCV-only system of Equations (28), (29), (30) in the form of Equation (3.1) of [28] and using the same notation as used in [28], we then have

(37) $$\begin{array}{c} X=\left(S_{\text{HCV}}\right),Z=\left(I_{C},C_{L}\right),F\left(X,0\right)=\left[\wedge -{\mu S}_{\text{HCV}}\right], \end{array}$$

and

(38) $$\begin{array}{c} A=\left[\begin{array}{cc} \tilde{c}\beta_{2}-\left(\gamma +\tau +\mu \right) & \tilde{c}\beta_{2}\\ \gamma & -\left(\mu +\emptyset \right) \end{array}\right],\hat{G}\left(X,Z\right)=\left[\begin{array}{c} \tilde{c}\beta_{2}\left(I_{C}+C_{L}\right)\left(1-\frac{S_{\text{HCV}}}{N_{\text{HCV}}}\right)\\ 0 \end{array}\right]. \end{array}$$

Since 0 ≤ *S*_HCV_ ≤ *N*_HCV_, it is easy to see that $\hat{G}\left(X,Z\right)\geq 0$. We also notice that for matrix  *A*, element *a*_11_ < 0 when $\tilde{c}\beta_{2}<\left(\gamma +\tau +\mu \right)$, which implies that it is an *M*-matrix since all its off diagonal elements are nonnegative. Hence, *ε*_HCV_^0^  is globally asymptotically stable for *R*_HCV_ < 1.

#### 3.2.7. HCV Endemic Equilibrium

Here, we make an insight into the persistence of HCV. The HCV endemic equilibrium is given by

(39) $$\begin{array}{c} \varepsilon_{\text{HCV}}^{*}=\left(S_{\text{HCV},}^{*}I_{C}^{*},C_{L}^{*}\;\right)=\left(\frac{\wedge \left(\gamma +\tau +\mu \right)\left(\mu +\emptyset +\gamma \right)}{D},\frac{E}{D},\frac{\gamma E}{\left(\mu +\emptyset \right)D}\right), \end{array}$$

where $D=\left[\tilde{c}\beta_{2}\left(\mu +\emptyset +\gamma \right)\left(\gamma +\mu \right)-\gamma \emptyset \left(\gamma +\tau +\mu \right)\right]$and

(40) $$\begin{array}{c} E=\wedge \left[\tilde{c}\beta_{2}\left(\mu +\emptyset +\gamma \right)-\left(\mu +\emptyset \right)\left(\gamma +\tau +\mu \right)\right]. \end{array}$$

**Lemma 4 .**

HCV endemic equilibrium, *ε*_HCV_^∗^, is locally asymptotically stable if R_HCV_ > 1 otherwise unstable.

**Proof**

In a similar argument as Lemma 1, the proof goes through to show that

(41) $$\begin{array}{c} \text{tr}\left(J\left(\varepsilon_{\text{HCV}}^{*}\right)\right)<0\text{ when }\frac{R_{\text{HCV}}\left(\tilde{c}\beta_{2}+3\mu +\left(\gamma +\tau +\mu \right)\right)}{2\tilde{c}\beta_{2}}>1,\\ \det\left(J\left(\varepsilon_{\text{HCV}}^{*}\right)\right)>0\text{ if }\frac{\tilde{c}\beta_{2}\left(\mu +\emptyset +\gamma \right)}{\left(\mu +\emptyset \right)\left(\gamma +\tau +\mu \right)}=R_{\text{HCV}}<1. \end{array}$$

Since det(*J*(*ε*_HCV_^∗^)) is positive when *R*_HCV_ < 1, then, HCV endemic equilibrium, *ε*_HCV_^∗^, is locally asymptotically unstable.

#### 3.2.8. Global Stability of HCV Endemic Equilibrium for HCV-Only Submodel

To investigate the global stability of *ε*_HCV_^∗^, we proceed like in Subsection 3.2.4.

**Lemma 5 .**

If  *R*_HCV_ > 1, then the HCV endemic equilibrium of HCV-only submodel, *ε*_HCV_^∗^, is globally asymptotically stable.

**Proof**

We define the Lyapunov function, *Q* = *Q*(*S*_*HCV*_, *I*_*C*_, *C*_*L*_), as

(42) $$\begin{array}{c} Q=U_{1}\left(S_{\text{HCV}}-S_{\text{HCV}}^{*}-S_{\text{HCV}}^{*}\text{In}\left(\frac{S_{\text{HCV}}}{S_{\text{HCV}}^{*}}\right)\right)+U_{2}\left(I_{C}-I_{C}^{*}-I_{C}^{*}\text{In}\left(\frac{I_{C}}{I_{C}^{*}}\right)\right)+U_{3}\left(C_{L}-C_{L}^{*}-C_{L}^{*}\text{In}\left(\frac{C_{L}}{C_{L}^{*}}\right)\right). \end{array}$$

The time derivative of the Lyapunouv function is given by

(43) $$\begin{array}{c} \frac{dQ}{dt}=U_{1}\left(1-\frac{S_{\text{HCV}}^{*}}{S_{\text{HCV}}}\right)\frac{dS_{\text{HCV}}}{dt}+U_{2}\left(1-\frac{I_{C}^{*}}{I_{C}}\right)\frac{dI_{C}}{dt}+U_{3}\left(1-\frac{C_{L}^{*}}{C_{L}}\right)\frac{dC_{L}}{dt}. \end{array}$$

Following a similar argument as in the proof of Lemma 2, we have

(44) $$\begin{array}{c} \frac{dQ}{dt}=-B+H, \end{array}$$

where

(45) $$\begin{array}{c} B=U_{1}\frac{{\left(S_{\text{HCV}}-S_{\text{HCV}}^{*}\right)}^{2}}{S_{\text{HCV}}}\mu +U_{1}\tau I_{C}^{*}+U_{1}\frac{\tau I_{C}S_{\text{HCV}}^{*}}{S_{\text{HCV}}}+U_{1}\frac{\tilde{c}\beta_{2}S_{\text{HCV}}^{*}I_{C}^{*}}{N_{\text{HCV}}^{*}}\frac{S_{\text{HCV}}^{*}}{S_{\text{HCV}}}+U_{1}\frac{\tilde{c}\beta_{2}S_{\text{HCV}}^{*}C_{L}^{*}}{N_{\text{HCV}}^{*}}\frac{S_{\text{HCV}}^{*}}{S_{\text{HCV}}}+U_{1}\frac{\tilde{c}\beta_{2}S_{\text{HCV}}I_{C}}{N_{\text{HCV}}}+U_{1}\frac{\tilde{c}\beta_{2}S_{\text{HCV}}C_{L}}{N_{\text{HCV}}}+U_{2}\frac{\tilde{c}\beta_{2}S_{\text{HCV}}I_{C}^{*}}{N_{\text{HCV}}}+U_{2}\frac{\tilde{c}\beta_{2}C_{L}S_{\text{HCV}}I_{C}^{*}}{N_{\text{HCV}}I_{C}}+U_{2}\frac{\tilde{c}\beta_{2}S_{\text{HCV}}^{*}I_{C}}{N_{\text{HCV}}^{*}}+U_{2}\frac{\tilde{c}\beta_{2}C_{L}^{*}S_{\text{HCV}}^{*}I_{C}}{N_{\text{HCV}}^{*}I_{C}^{*}}+U_{3}\frac{\gamma C_{L}^{*}I_{C}}{C_{L}}+U_{3}\frac{\gamma C_{L}I_{C}^{*}}{C_{L}^{*}}, \end{array}$$

and

(46) $$\begin{array}{c} H=U_{1}\frac{\tau I_{C}^{*}S_{\text{HCV}}^{*}}{S_{\text{HCV}}}+U_{1}\tau I_{C}+U_{1}\frac{\tilde{c}\beta_{2}S_{\text{HCV}}^{*}I_{C}^{*}}{N_{\text{HCV}}^{*}}+U_{1}\frac{\tilde{c}\beta_{2}S_{\text{HCV}}^{*}C_{L}^{*}}{N_{\text{HCV}}^{*}}+U_{1}\frac{\tilde{c}\beta_{2}S_{\text{HCV}}^{*}I_{C}}{N_{\text{HCV}}}+U_{1}\frac{\tilde{c}\beta_{2}S_{\text{HCV}}^{*}C_{L}}{N_{\text{HCV}}}+U_{2}\frac{\tilde{c}\beta_{2}S_{\text{HCV}}I_{C}}{N_{\text{HCV}}}+U_{2}\frac{\tilde{c}\beta_{2}S_{\text{HCV}}C_{L}}{N_{\text{HCV}}}+U_{2}\frac{\tilde{c}\beta_{2}S_{\text{HCV}}^{*}I_{C}^{*}}{N_{\text{HCV}}^{*}}+U_{2}\frac{\tilde{c}\beta_{2}S_{\text{HCV}}^{*}C_{L}^{*}}{N_{\text{HCV}}^{*}}+U_{3}\gamma I_{C}+U_{3}\gamma I_{C}^{*}. \end{array}$$

Hence, HCV endemic equilibrium, *ε*_HCV_^∗^, is globally asymptotically stable if *H* < *B*.

#### 3.2.9. The Disease-Free Equilibrium and Reproduction Number for the HIV-HCV Coinfection Model

HIV-HCV coinfection model has a disease-free equilibrium, given by *ε*^0^ = (*S*^0*f*^, *I*_*H*_^0*f*^, *I*_*C*_^0*f*^, *I*_*HC*_^0*f*^, *I*_*HC*_*L*__^0*f*^, *C*_*L*_^0*f*^) = (∧/*μ*, 0, 0, 0, 0, 0).

**Lemma 6 .**

The basic reproduction number for HIV-HCV coinfection model

(47) $$\begin{array}{c} R_{0}=\max\left\{R_{\text{HIV}},R_{\text{HCV}}\right\}. \end{array}$$

**Proof**

Using the next generation matrix method of computing the basic reproduction number [27] on model Equations (3), (4), (5), (6), (7), and (8), we obtain Jacobian of new infection matrix at disease free-equilibrium, $\tilde{F}$, as

(48) $$\begin{array}{c} \tilde{F}=\left[\begin{array}{ccccc} \tilde{c}\beta_{1} & 0 & \tilde{c}\beta_{1}\omega & \tilde{c}\beta_{1}\omega & 0\\ 0 & \tilde{c}\beta_{2} & \tilde{c}\beta_{2}\rho & \tilde{c}\beta_{2}\rho & \tilde{c}\beta_{2}\\ 0 & 0 & 0 & 0 & 0\\ 0 & 0 & 0 & 0 & 0\\ 0 & 0 & 0 & 0 & 0 \end{array}\right] \end{array}$$

and the Jacobian of the matrix for transfer from one compartment to another at disease-free

equilibrium, *V*, as

(49) $$\begin{array}{c} V=\left[\begin{array}{ccccc} \left(\alpha +\mu \right) & 0 & -r\tau & 0 & 0\\ 0 & \left(\gamma +\tau +\mu \right) & 0 & 0 & 0\\ 0 & 0 & \left(\mu +r\tau +\theta \right) & 0 & 0\\ 0 & 0 & -\theta & \mu & 0\\ 0 & -\gamma & 0 & 0 & \left(\mu +\emptyset \right) \end{array}\right]. \end{array}$$

The basic reproduction number, *R*_0_, for the HIV-HCV coinfection model is the maximum of eigenvalues *λ*_1_, *λ*_2_, *λ*_3_, *λ*_4_, and *λ*_5_ of the next generation matrix, $\tilde{F}V^{-1}$.

That is, $R_{0}=\max\left\{\left(\tilde{c}\beta_{1}/\left(\alpha +\mu \right)\right),\left(\tilde{c}\beta_{2}/\left(\gamma +\tau +\mu \right)\right)\left(1+\left(\gamma /\left(\mu +\emptyset \right)\right)\right),0,0,0\right\}$.

Thus,

(50) $$\begin{array}{c} R_{0}=\max\left\{R_{\text{HIV}},R_{\text{HCV}}\right\}, \end{array}$$

where *R*_HIV_ and *R*_HCV_ are the basic reproduction numbers of HIV-only and HCV-only submodels as indicated in Equations (18) and (33), respectively.

This implies that the dynamics of the HIV-HCV coinfection will be dominated by the disease with the bigger basic reproduction number.

**Lemma 7 .**

The disease-free equilibrium, *ε*^0^, of the HIV-HCV coinfection model is locally asymptotically stable if *R*_0_ < 1 and unstable otherwise.

**Proof**

The disease-free equilibrium is locally asymptotically stable if and only if all the roots of *J*_11_  and *J*_22_  have negative real parts [31, 32]. The Jacobian matrix of the HIV-HCV coinfection model at  *ε*^0^,  *J*(*ε*^0^), is given by

(51) $$\begin{array}{c} \left[\begin{array}{cccccc} -\mu & -\tilde{c}\beta_{1} & \tau -\tilde{c}\beta_{2} & -\left(\tilde{c}\beta_{1}\omega +\tilde{c}\beta_{2}\rho \right) & -\left(\tilde{c}\beta_{1}\omega +\tilde{c}\beta_{2}\rho \right) & -\tilde{c}\beta_{2}\\ 0 & \tilde{c}\beta_{1}-\left(\alpha +\mu \right) & 0 & \tilde{c}\beta_{1}\omega +r\tau & \tilde{c}\beta_{1}\omega & 0\\ 0 & 0 & \tilde{c}\beta_{2}-\left(\gamma +\tau +\mu \right) & \tilde{c}\beta_{2}\rho & \tilde{c}\beta_{2}\rho & \tilde{c}\beta_{2}\\ 0 & 0 & 0 & -\left(\mu +r\tau +\theta \right) & 0 & 0\\ 0 & 0 & 0 & \theta & -\mu & 0\\ 0 & 0 & \gamma & 0 & 0 & -\left(\mu +\emptyset \right) \end{array}\;\right] \end{array}$$

Now, we rewrite  *J*(*ε*^0^) as

(52) $$\begin{array}{c} J\left(\varepsilon^{0}\right)=\left[\begin{array}{cc} J_{11} & J_{12}\\ J_{21} & J_{22} \end{array}\right], \end{array}$$

where

(53) $$\begin{array}{c} J_{11}=\left[\begin{array}{ccc} -\mu & -\tilde{c}\beta_{1} & \tau -\tilde{c}\beta_{2}\\ 0 & \tilde{c}\beta_{1}-\left(\alpha +\mu \right) & 0\\ 0 & 0 & \tilde{c}\beta_{2}-\left(\gamma +\tau +\mu \right) \end{array}\right],J_{12}=\left[\begin{array}{ccc} -\left(\tilde{c}\beta_{1}\omega +\tilde{c}\beta_{2}\rho \right) & -\left(\tilde{c}\beta_{1}\omega +\tilde{c}\beta_{2}\rho \right) & -\tilde{c}\beta_{2}\\ \tilde{c}\beta_{1}\omega +r\tau & \tilde{c}\beta_{1}\omega & 0\\ \tilde{c}\beta_{2}\rho & \tilde{c}\beta_{2}\rho & \tilde{c}\beta_{2} \end{array}\right],J_{21}=\left[\begin{array}{ccc} 0 & 0 & 0\\ 0 & 0 & 0\\ 0 & 0 & \gamma \end{array}\right],J_{22}=\left[\begin{array}{ccc} -\left(\mu +r\tau +\theta \right) & 0 & 0\\ \theta & -\mu & 0\\ 0 & 0 & -\left(\mu +\emptyset \right) \end{array}\right]. \end{array}$$

The eigenvalues of *J*_22_ are −*μ*, −(*μ*+∅), and −(*μ* + *rτ* + *θ*) which are all negative.

The eigenvalues of *J*_11_ are $-\mu ,\tilde{c}\beta_{1}-\left(\alpha +\mu \right),$ and $\tilde{c}\beta_{2}-\left(\gamma +\tau +\mu \right)$. However, all the eigenvalues of *J*_11_ are negative when

(54) $$\begin{array}{c} \tilde{c}\beta_{1}<\left(\alpha +\mu \right)\text{ from which }R_{\text{HIV}}=\frac{\tilde{c}\beta_{1}}{\left(\alpha +\mu \right)}<1, \end{array}$$

and

(55) $$\begin{array}{c} \tilde{c}\beta_{2}<\left(\gamma +\tau +\mu \right)\text{ from which }\frac{\tilde{c}\beta_{2}}{\left(\gamma +\tau +\mu \right)}=\frac{\left(\mu +\emptyset \right)R_{\text{HCV}}}{\left(\mu +\emptyset +\gamma \right)}<1 \end{array}$$

Therefore, if inequalities in (54) and (55) are satisfied, then *R*_0_ < 1.

#### 3.2.10. Global Stability of Disease-Free Equilibrium for HIV-HCV Coinfection Model

We proceed like in Subsections 3.2.2 and 3.2.6. Rewriting HIV-HCV coinfection model Equations (3), (4), (5), (6), (7), and (8) in the form of Equation (3.1) of [28] and using the same notation as used in [28], we have

(56) $$\begin{array}{c} X=\left(S\right),Z=\left(I_{H},I_{C},I_{\text{HC}},I_{{\text{HC}}_{L}},C_{L}\right),F\left(X,0\right)=\left[\wedge -\mu S\right], \end{array}$$

and

(57) $$\begin{array}{c} A=\left[\;\begin{array}{ccccc} \tilde{c}\beta_{1}-\left(\alpha +\mu \right) & 0 & \tilde{c}\beta_{1}\omega +r\tau & \tilde{c}\beta_{1}\omega & 0\\ 0 & \tilde{c}\beta_{2}-\left(\gamma +\tau +\mu \right) & \tilde{c}\beta_{2}\rho & \tilde{c}\beta_{2}\rho & \tilde{c}\beta_{2}\\ 0 & 0 & -\left(\mu +r\tau +\theta \right) & 0 & 0\\ 0 & 0 & \theta & -\mu & 0\\ 0 & \gamma & 0 & 0 & -\left(\mu +\emptyset \right) \end{array}\;\right],\\ \hat{G}\left(X,Z\right)=\left[\begin{array}{c} {\hat{G}}_{1}\left(X,Z\right)\\ {\hat{G}}_{2}\left(X,Z\right)\\ {\hat{G}}_{3}\left(X,Z\right)\\ {\hat{G}}_{4}\left(X,Z\right)\\ {\hat{G}}_{5}\left(X,Z\right) \end{array}\right]=\left[\begin{array}{c} \tilde{c}\beta_{1}\left(1-\frac{S}{N}\right)\left(I_{H}+\omega I_{\text{HC}}+\omega I_{{\text{HC}}_{L}}\right)+k_{2}\pi_{C}I_{H}\\ \tilde{c}\beta_{2}\left(1-\frac{S}{N}\right)\left(I_{C}+C_{L}+\rho I_{\text{HC}}+\rho I_{{\text{HC}}_{L}}\right)+k_{1}\pi_{H}I_{C}\\ -k_{2}\pi_{C}I_{H}-k_{1}\pi_{H}I_{C}\\ -k_{3}\pi_{H}C_{L}\\ k_{3}\pi_{H}C_{L} \end{array}\right]. \end{array}$$

Since ${\hat{G}}_{3}\left(X,Z\right)<0\;$ and ${\hat{G}}_{4}\left(X,Z\right)<0$, then $\hat{G}\left(X,Z\right)<0$, an implication that the second condition (*H*_2_)  in Theorem by [28] is not satisfied. Consequently, the disease-free equilibrium of HIV-HCV coinfection system is not globally asymptotically stable for *R*_0_ < 1.

#### 3.2.11. HIV-HCV Coinfection Endemic Equilibrium

**Figure 2:**
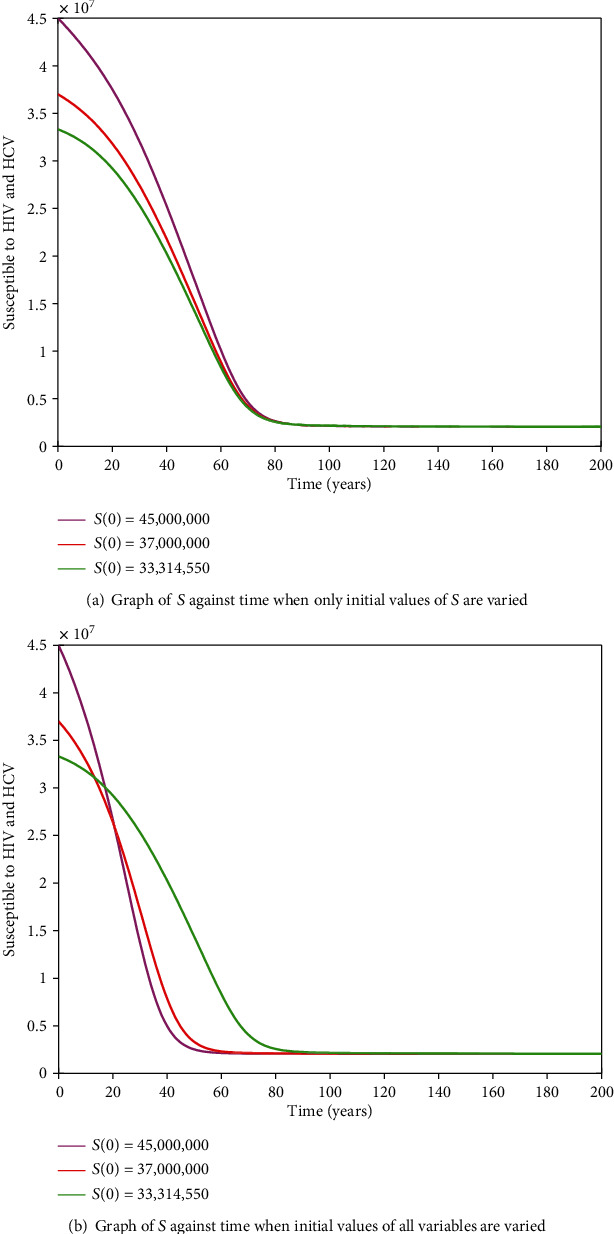
The dynamics of individuals susceptible to HCV and HIV, *S*, under different initial conditions. Irrespective of the different initial conditions, in the long run, the number susceptible to HIV and HCV infections is the same.

**Figure 3:**
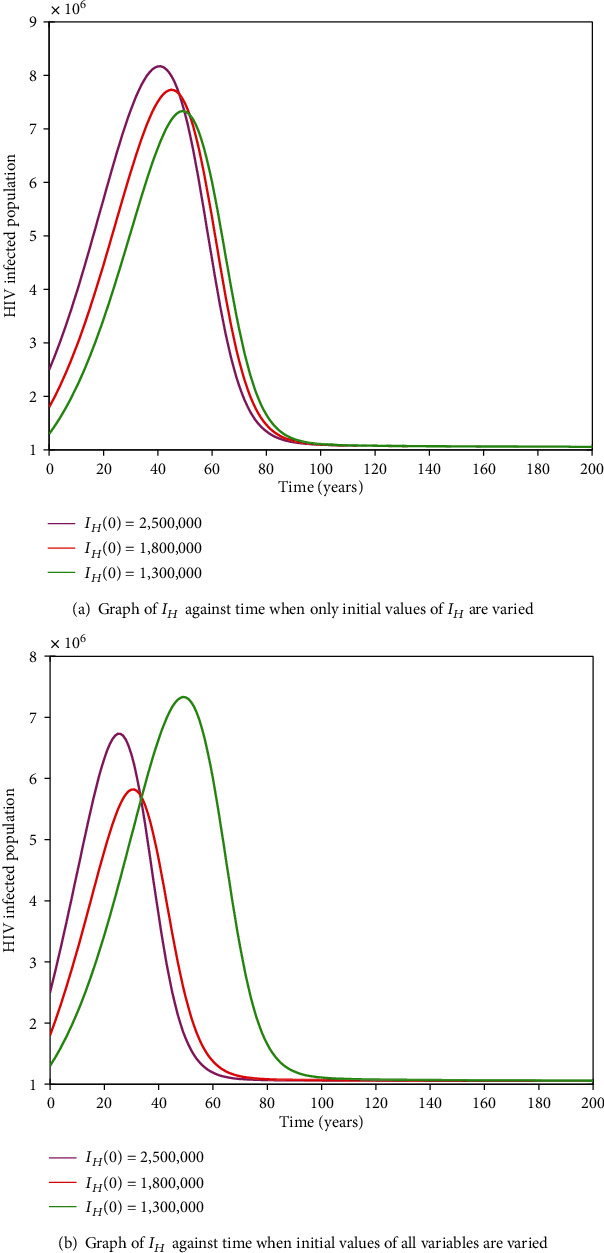
The dynamics of HIV-infected individuals, *I*_*H*_, under different initial conditions. In the long run, irrespective of the different initial conditions, the number of HIV-infected individuals is the same.

**Figure 4:**
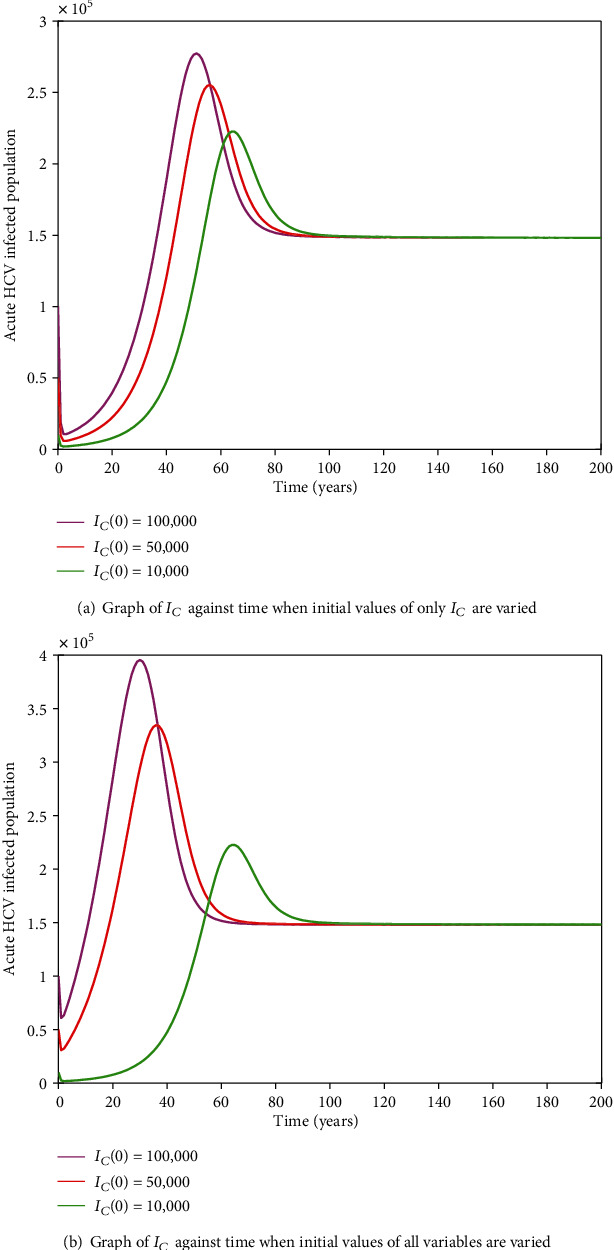
The dynamics of acute HCV-infected individuals, *I*_*C*_, under different initial conditions. In the long run, the number of acute HCV-infected individuals is the same for the different initial conditions.

**Figure 5:**
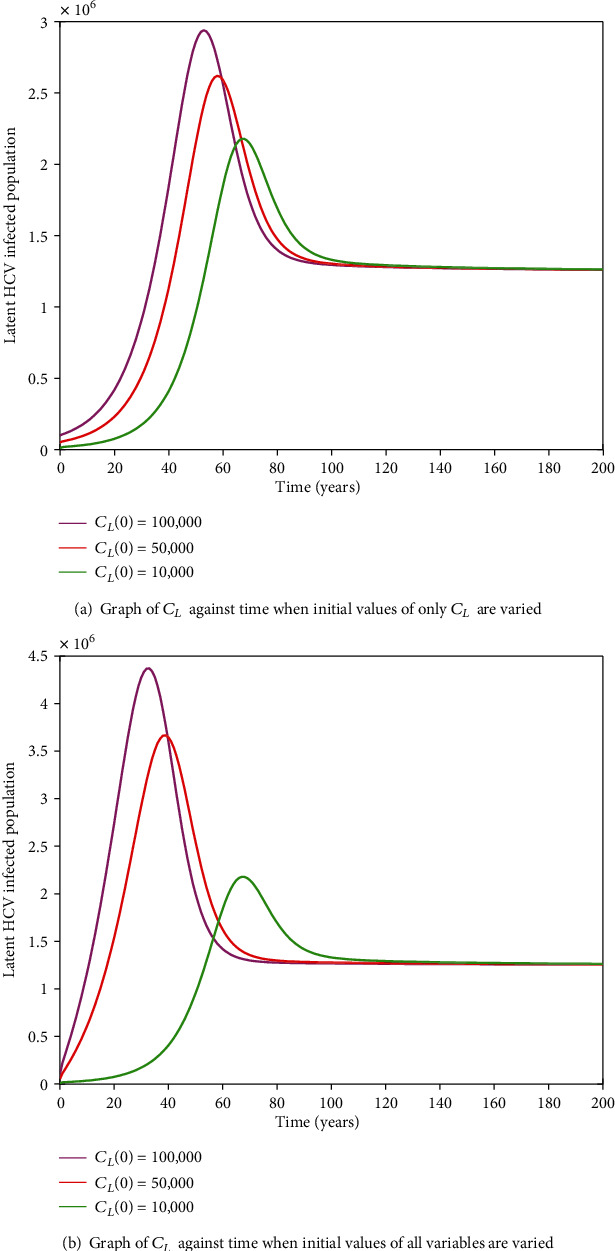
The dynamics of latent HCV-infected individuals, *C*_*L*_, under different initial conditions. In the long run, the number of *C*_*L*_  is the same for different initial conditions.

**Figure 6:**
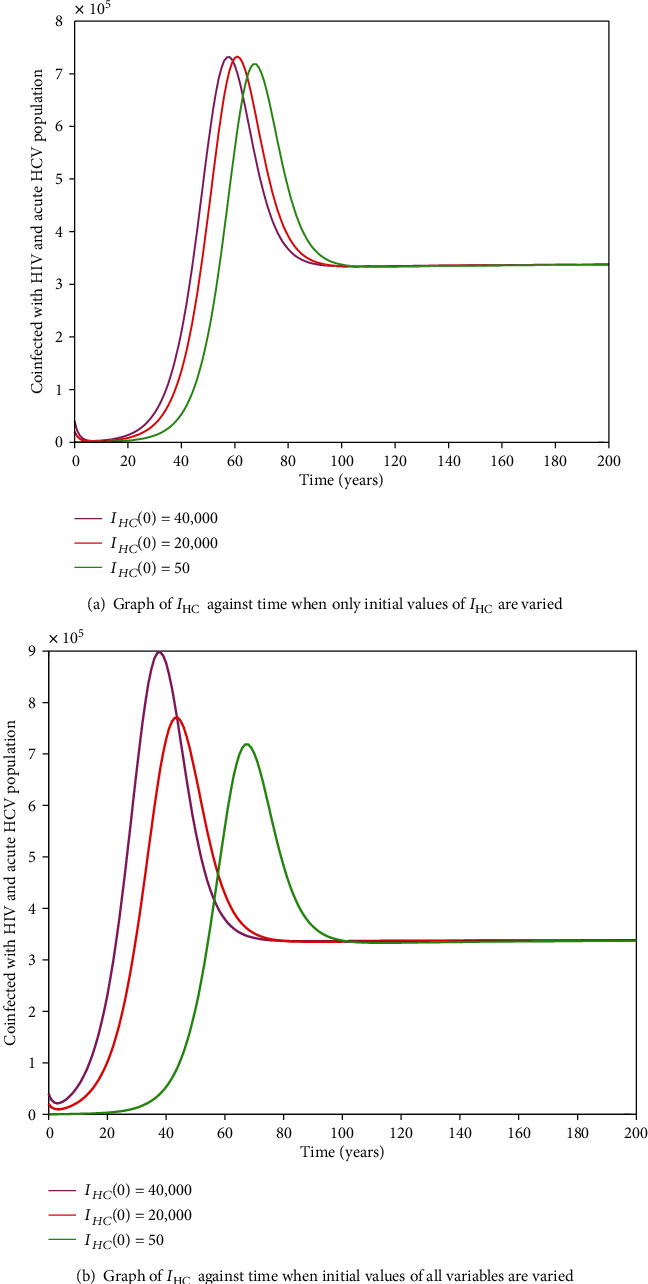
The dynamics of individuals coinfected with HIV and acute HCV, *I*_HC_, under different initial conditions. The number of *I*_HC_ is the same for different initial conditions.

**Figure 7:**
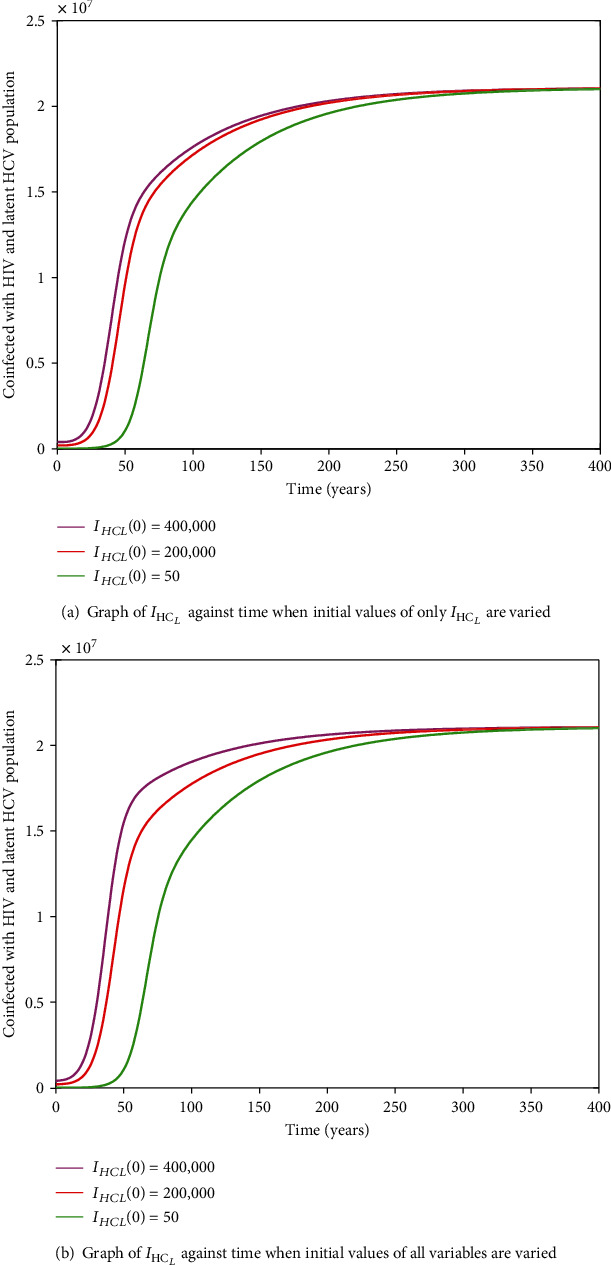
The dynamics of individuals coinfected with HIV and latent HCV, *I*_HC_*L*__, under different initial conditions. In the long run, the number of HIV and latent HCV-coinfected individuals is the same, irrespective of the different initial conditions.

It is cumbersome to analytically establish expressions for the endemic equilibrium for the HIV-HCV coinfection model. We hereby numerically investigate its existence and stability. This is done by varying the initial values of the variables to determine whether they would level off to the same nonzero values in the long run, irrespective of the different initial values of the variables. Furthermore, values of some of arbitrarily selected parameters are varied to determine whether some variables, arbitrarily selected, would level off to nonzero values in the long run. Figures 2(a) and 2(b)–7(a) and 7(b) show the existence of a stable endemic equilibrium and the nonexistence of the stable disease-free equilibrium of the HIV-HCV coinfection model. All the graphs of susceptible to HIV and HCV against time, HIV-infected population against time, acute HCV-infected population against time, latent HCV-infected population against time, HIV and acute HCV-coinfected population against time, and HIV and latent HCV-coinfected population against time finally attain a nonzero steady state as indicated in Figures 2–7, respectively.

In all these simulations, the values of parameters used are as presented in Table 1. In each of the Figures 2(a), 3(a), 4(a), 5(a), 6(a), and 7(a), respectively, initial sizes of individuals who are susceptible to HIV and HCV infections, *S*(0); HIV infected, *I*_*H*_(0); acute HCV infected, *I*_*C*_(0); latent HCV infected, *C*_*L*_(0); HIV and acute HCV coinfected, *I*_*HC*_(0); and HIV and latent HCV coinfected, *I*_*HC*_*L*__(0) are varied while keeping initial values of the other variables constant.

Figure 2(a) reveals that in the long run, irrespective of the initial value of individuals susceptible to HIV and HCV infections, the number that is left susceptible to HIV and HCV infections is the same. For the HIV-infected individuals, the number of HIV infections in the long run is the same for the different initial values *I*_*H*_(0) as shown in Figure 3(a). For the acute HCV infected individuals, in the long run, the number of acute HCV-infected individuals is the same for the different initial values *I*_*C*_(0) as shown in Figure 4(a). In the long run, the number of latent HCV-infected individuals is the same for the different initial values *C*_*L*_(0) as shown in Figure 5(a). In the long run, irrespective of the initial values  *I*_*HC*_(0), the number of HIV and acute HCV-coinfected individuals is the same as shown in Figure 6(a). The number of HIV and latent HCV-coinfected individuals is the same for the different initial values *I*_*HC*_*L*__(0)  as shown in Figure 7(a).

In Figures 2(b), 3(b), 4(b), 5(b), 6(b), and 7(b), respectively, initial values of the indicated variable are varied simultaneously with varying initial values of the rest of the other variables of the HIV-HCV coinfection model. It is revealed that in the long run, irrespective of the different initial values of all the variables of the HIV-HCV coinfection model, all the graphs level off to nonzero values as it is the case with the respective corresponding Figure (a). Hence, there exists a globally stable endemic equilibrium for HIV-HCV coinfection model.

**Figure 8:**
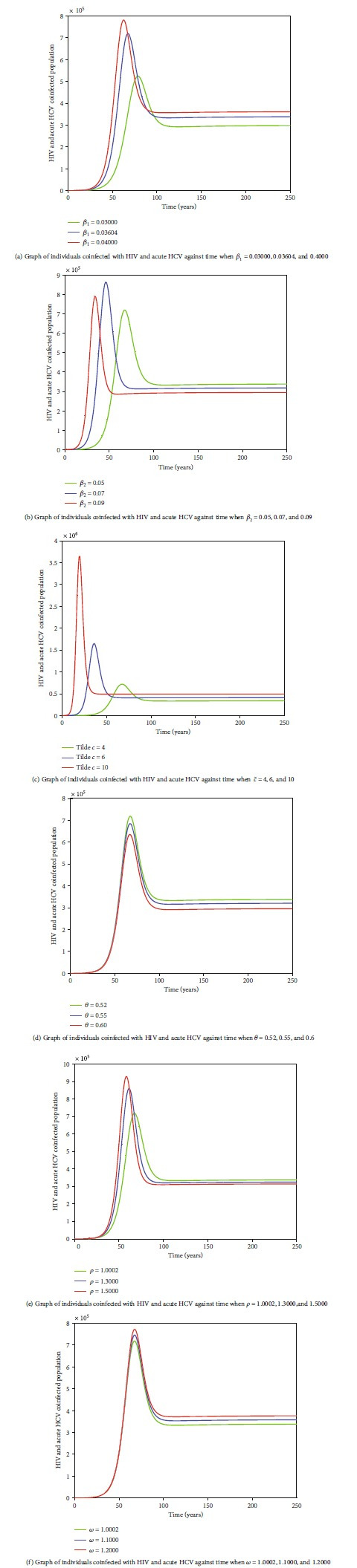
The dynamics of individuals coinfected with HIV and acute HCV, *I*_HC_, under varying values of *β*_1_, *β*_2_, $\tilde{c}$, *θ*, *ρ*, and *ω*. In the long run, all graphs level off to nonzero values of *I*_HC_, irrespective of the different values of the respective parameter.

**Figure 9:**
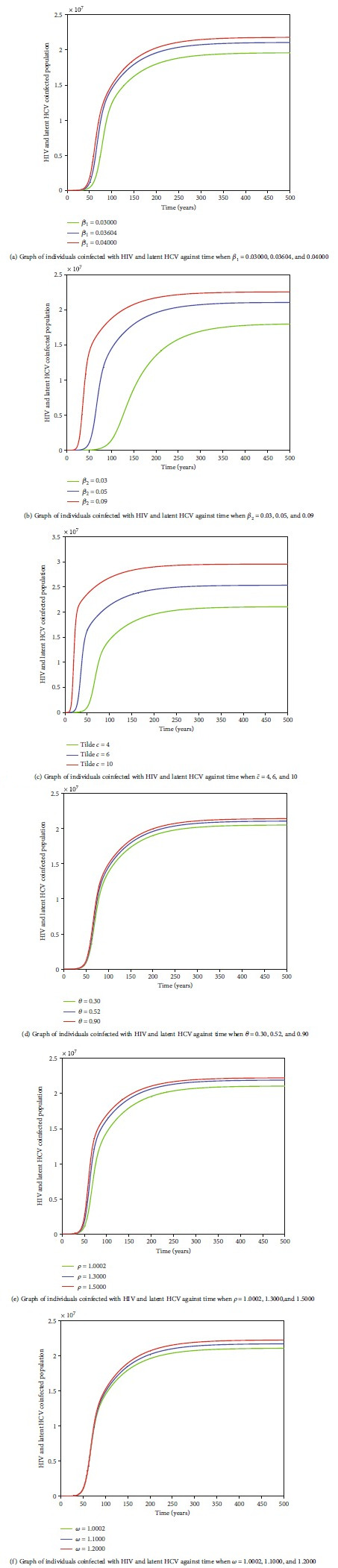
The dynamics of individuals coinfected with HIV and latent HCV, *I*_HC_*L*__, under varying values of parameters *β*_1_, *β*_2_, $\tilde{c}$, *θ*, *ρ*, and *ω*. In the long run, all graphs level off to nonzero values of *I*_HC_*L*__, irrespective of the different values of the respective parameter.

Figures 8(a)–8(f) and Figures 9(a)–9(f) also reveal an existence of a globally stable endemic equilibrium and nonexistence of stable disease-free equilibrium for HIV-HCV coinfection model. Values of arbitrarily selected parameters, namely, HIV transmission probability per sexual contact, *β*_1_; HCV transmission probability per sexual contact, *β*_2_; average number of sexual partners acquired per year, $\tilde{c}$; rate of progression from *I*_HC_ to *I*_HC_*L*__  class, *θ*; enhancement factor for increased risk of being infected with HCV by an HIV-HCV-coinfected individual, *ρ*; and enhancement factor for increased risk of being infected with HIV by an HIV-HCV-coinfected individual, *ω* were varied for some arbitrarily selected variables, namely, coinfected with HIV and acute HCV, *I*_*HC*_ and coinfected with HIV and latent HCV, *I*_*HC*_*L*__, to determine whether these variables would level off to nonzero values in the long run. Figures 8(a)–8(f) and 9(a)–9(f) reveal that irrespective of the different values of *β*_1_, *β*_2_, $\tilde{c}$, *θ*, *ρ*, and *ω*, in the long run, *I*_HC_ and *I*_HC_*L*__  attain a nonzero steady state, hence an existence of a globally stable endemic equilibrium for HIV-HCV coinfection model.

### 3.3. Sensitivity Analysis

#### 3.3.1. Derivation of Parameter Values

The recruitment rate of individuals into the susceptible class, ∧ , has been calculated using the expression ∧ = *φe*^−*μa*^, where *a*  is the age at first sexual debut (age at which people become sexually active); *φ* is the number of people that would become of *a* years of age, which is 16 years [24], and *μ* is the natural death rate. All the data used in calculating ∧ were obtained from Uganda. Now, using life expectancy as of 2014, which was estimated as 63.3 years [33], the natural mortality rate in Uganda is calculated as

(58) $$\begin{array}{c} \mu =\frac{1}{\text{life expectance}}=\frac{1}{63.3\text{ years}}=0.0158\;{\text{year}}^{-1}. \end{array}$$

In Uganda, 16 years ago (that is, in 2002), the total population was 24,227,297 with growth rate of 3.2% [33]. Assuming there was no death, the number of children that were born in 2002 that would become sexually active in 2019 would be

(59) $$\begin{array}{c} \frac{3.2}{100}\times 24,227,297=775,273.504. \end{array}$$

This implies that *φ* = 775,273.504. Probability of survival to age *a*  is *e*^−*μa*^ = *e*^−0.0158×16^.

Therefore, the number that sexually mature, ∧, is given by

(60) $$\begin{array}{c} \wedge =\varphi e^{-\mu a}=775,273.504\times e^{-0.0158\times 16}=602,095. \end{array}$$

Average number of sexual partners acquired per year, $\tilde{c}$, has been derived from the study of Renzaho et al. [24]. It was found out that the average number of sexual partners in the last 12 months preceding the survey was in the range 1 to 4. The data were collected on participants aged between 13 and 24 years. Since the reasons why a person in the age group of 13 and 24 years would go for more than one sexual partners are the same for any person who is sexually active, this study uses $\tilde{c}=4\;{\text{year}}^{-1}$. Rate of progression of infected individuals from acute to latent HCV (*γ*) has been derived from Sanchez et al. [18]. According to [18], duration in acute infection stage of HCV was 4 to 6 months. In this study, 6 months (0.5 years) are used as the duration. This implies that, *γ* = 1/0.5 years = 2 year^−1^. HIV transmission probability per sexual contact (*β*_1_ = 0.03604) has been derived from the study of Pinkerton [34]. Pinkerton estimated the per-act transmission probability during acute infection as 0.03604.

Using MATLAB, the following parameter values have been assumed only to illustrate the numerical results: rate of progression of individuals who are dually infected with HIV and acute HCV to dually infected with HIV and latent HCV, *θ*; rate of progression of individuals from latent HCV to advanced HCV, ∅; and rate of progression of individuals infected with HIV to AIDS, *α*. In addition, the following parameter values are cited from the respective studies with literature similar to this work as indicated in Table 1: amplification factors (*k*_*i*_, *i* = 1, 2, 3); HCV transmission probability per sexual contact, *β*_2_; enhancement factor for increased risk of being infected with HIV (*ω*) or HCV (*ρ*) by a dually infected individual; rate of spontaneous clearance of acute HCV, *τ*; and reduction factor for risk of acute HCV spontaneous clearance, *r*. The derived values of these parameters are presented in Table 1.

Substituting for the parameter values in Table 1 in (18) and (33), values of *R*_HIV_ = 1.680 and *R*_HCV_ = 1.667 are obtained. From (50), the basic reproduction number of the HIV-HCV coinfection model is obtained as *R*_0_ = max{*R*_HIV_, *R*_HCV_} = max{1.680, 1.667} = 1.680. Since *R*_HIV_ > *R*_HCV_, this confirms that the dynamics of HIV-HCV coinfection is dominated by HIV.

#### 3.3.2. Computation of Sensitivity Indices of the Basic Reproduction Number with Respect to the Parameters of the Model

In order to determine how best to reduce human mortality and morbidity due to HIV and HCV infections, it essentially requires knowledge of the relative importance of the different factors responsible for their transmission and prevalence. In this subsection, sensitivity analysis is carried out to determine the robustness of parameters that have high impact on the basic reproduction number, *R*_0_, such that appropriate intervention strategies can be taken. This is achieved by computing sensitivity indices of the basic reproduction number with respect to the parameters of the model using the normalized forward sensitivity index method [35]. The normalized forward sensitivity index of a variable, *R*_*e*_, that depends on a parameter, *x*, is defined as the ratio of the relative change in *R*_*e*_  to the relative change in parameter, *x*, that is

(61) $$\begin{array}{c} r_{x}^{R_{e}}=\frac{\partial R_{e}}{\partial x}\times \frac{x}{R_{e}}. \end{array}$$

Since *R*_0_ = max{*R*_HIV_, *R*_HCV_}, the sensitivity analysis of *R*_0_ with respect to each of the parameters has been discussed via the sensitivities of *R*_HIV_  and *R*_HCV_. This implies that the parameters of interest will largely depend on the dominant disease. Sensitivity indices of *R*_HIV_  and *R*_HCV_  have been calculated analytically using formulas

(62) $$\begin{array}{c} r_{x}^{R_{\text{HIV}}}=\frac{\partial R_{\text{HIV}}}{\partial x}\times \frac{x}{R_{\text{HIV}}},r_{x}^{R_{\text{HCV}}}=\frac{\partial R_{\text{HCV}}}{\partial x}\times \frac{x}{R_{\text{HCV}}}, \end{array}$$

respectively.

Sensitivity indices of both *R*_HIV_  and  *R*_HCV_ are presented in Table 2 in which the parameters are ordered from most sensitive to least.

*(1) Interpretation of the Sensitivity Indices*. The sensitivity indices presented in Table 2 are interpreted as follows: for parameters that have positive indices, it implies that the corresponding basic reproduction number increases (decreases) with increase (decrease) in those parameters. Conversely, for parameters that have negative indices, it implies that the corresponding basic reproduction number decreases (increases) with increase (decrease) in those parameters. For example, increasing (decreasing) the value of HIV transmission probability per sexual contact, *β*_1_, by 10% while the rest of the parameter values are kept fixed, increases (decreases) the value of *R*_HIV_  by 10%. On the other hand, a 10% increase (decrease) in the value of the rate of progression of individuals infected with HIV to AIDS, *α*, while keeping the values of other parameters constant, decreases (increases) the value of *R*_HIV_  by 8.2%.

We deduce that endemicity of HIV infection increases when the values of *β*_1_ and $\tilde{c}$ are increased and or those of *α*  and *μ*  are decreased. The most sensitive parameters in HIV infection are $\tilde{c}\;$ and *β*_1_ (which are equally sensitive) followed by *α*. Therefore, interventions should target and concentrate on reducing the values of average number of sexual partners acquired per year, $\tilde{c}$, and HIV transmission probability per sexual contact, *β*_1_, since increasing rate of progression from HIV to AIDS, *α*, would imply fast progression to AIDS. This is not desirable from an HIV-infected individual's perspective as earlier mentioned in Subsection 3.2.1. Furthermore, we also deduce that endemicity of HCV infection increases when the values of  *β*_2_, $\tilde{c}$, and *γ*  are increased and or those of ∅, *τ*, and *μ* are decreased. This is in agreement with the literature [8–11] which reveals that the risk of sexual transmission of HCV increases with multiple sexual partners. The most sensitive parameters in HCV infection are $\tilde{c}\;$ and *β*_2_ (which are equally sensitive) followed by ∅. Therefore, interventions of reducing HCV infection should target and concentrate on reducing values of $\tilde{c}\;$ and  *β*_2_. Increasing the value of ∅ would imply fast progression from latent to advanced HCV, which is not desirable from an HCV-infected individual's perspective.

In Subsection 3.2.9, it was revealed that the dynamics of HIV-HCV coinfection is dominated by HIV. Therefore, *R*_0_  will be more sensitive to  *β*_1_, $\tilde{c}$, and *α* just like *R*_HIV_. Sensitivity analysis reveals that HIV (or HCV) transmission probability per sexual contact and average number of sexual partners acquired per year are equally likely to increase HIV (or HCV) infections. Furthermore, increments in the values of these parameters lead into other parameters increasing the HIV (or HCV) infection. Therefore, for reduced HIV (or HCV) infections, individuals need to greatly reduce on the rate of sexual partner acquisition and HIV (or HCV) transmission probability per sexual contact (that is, having safe sex, like using condoms, which does not expose them to infected blood). On the other hand, the need to mention is that HIV and latently HCV-infected individuals need to seek for early treatment. This will slow down the progression of HIV to AIDS and latent HCV to advanced HCV.

## 4. Numerical Simulations

In this section, we carry out numerical simulations of the HIV-HCV coinfection model to study the HIV-HCV coinfection dynamics in absence of treatment. Simulations are performed to illustrate some of the theoretical results obtained in this work. Our system is an initial value problem, well posed epidemiologically and mathematically as illustrated in the proofs of Theorems 1 and 2. We used ode45 to simulate our problem. ode45 is a Runge-Kutta (4,5) nonstiff one-step solver in Matlab. It has a good speed and it is accurate as well as stable. It is more efficient than the Euler method. It is easy to implement and very stable when compared to multistep methods. Despite of the fact that it requires relatively more computer time than multistep methods of comparable accuracy, its advantage of the relative simplicity and ease of use far outweighs the disadvantage of its relatively high computational cost. The parameter values that are used for numerical simulations are presented in Table 1 and the following initial conditions are used. In Uganda, the total population (*P*) in 2014 was 34,634,650 [33]. *I*_*H*_(0) = 1, 300, 000, which is the total population that was living with HIV in 2015 [36].

(63) $$\begin{array}{c} S\left(0\right)=P-I_{C}\left(0\right)-I_{H}\left(0\right)-I_{HC}\left(0\right)-I_{{HC}_{L}}\left(0\right)-C_{L}\left(0\right)=33,314,550, \end{array}$$

in which

(64) $$\begin{array}{c} I_{C}\left(0\right)=10,000, \end{array}$$

(65) $$\begin{array}{c} C_{L}\left(0\right)=10,000\;\left(\text{all assumed}\right). \end{array}$$

**Figure 10:**
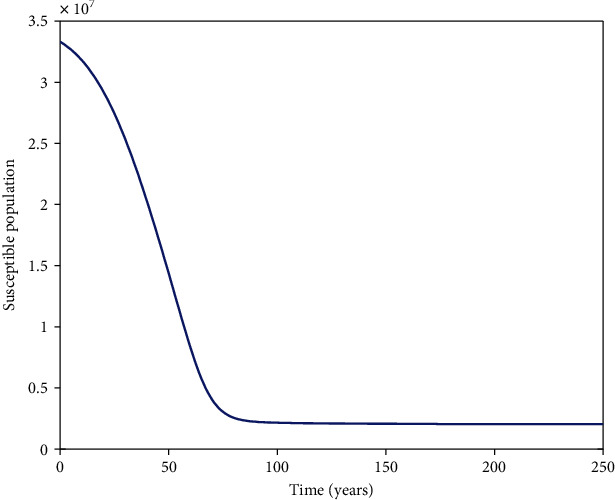
Simulation results showing a population susceptible to both HIV and HCV infections.

**Figure 11:**
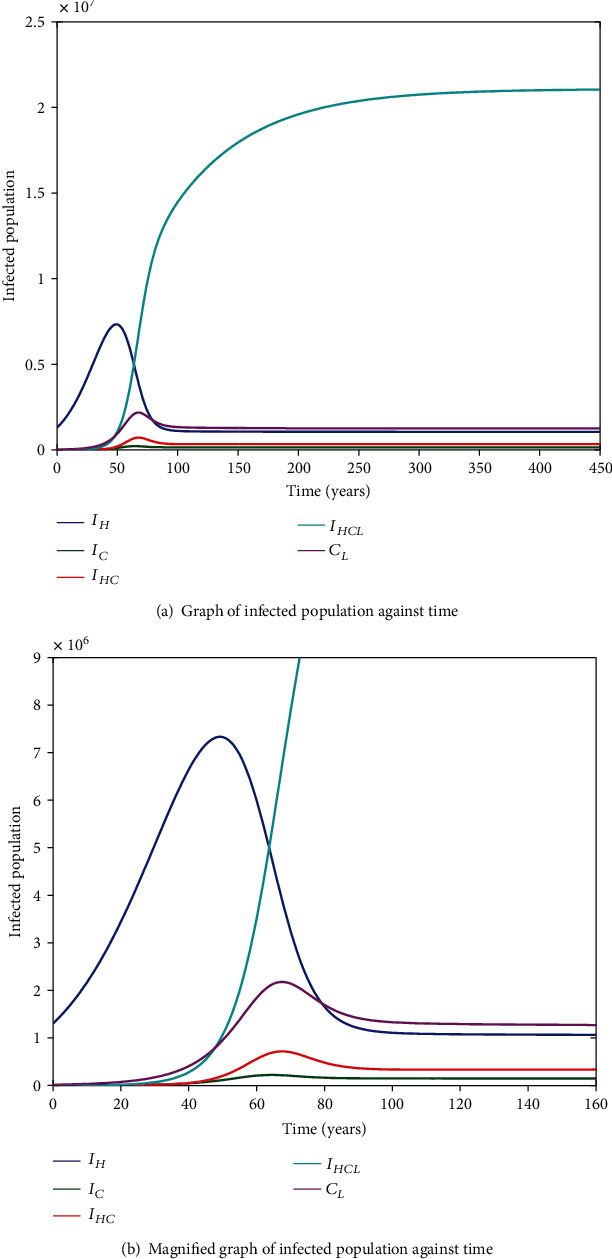
Simulation results showing HIV-HCV coinfection dynamics.

From Figure 10, we observe that in the absence of therapy for both HIV and HCV, the number of susceptibles reduces to their asymptotic state.  Figure 11(b) is a magnification of Figure 11(a). From Figures 11(a) and 11(b), it is noted that the number of individuals infected with HIV only (*I*_*H*_) start increasing and then in the long run it declines to steady state. In the long run, there are more individuals who are latently HCV infected (*C*_*L*_) as compared to those who are acutely HCV infected (*I*_*C*_). This is expected because the acute period is shorter than the latency stage. The number of individuals in *C*_*L*_ starts by increasing and in the long run it reduces to steady state. The number of individuals in *I*_*C*_ first increases and in the long run it decreases asymptotically to low levels. This is due to spontaneous clearance of acute HCV and other individuals acutely infected progressing to latent HCV. The number of individuals dually infected with HIV and acute HCV (*I*_HC_) starts increasing and in the long run it decreases asymptotically to low levels. The number of individuals dually infected with HIV and latent HCV (*I*_HC_*L*__) in the long run is more than that of individuals dually infected with HIV and acute HCV (*I*_HC_). The number of individuals in class *I*_HC_*L*__  increases to very high levels and it does not stop before attaining the steady state. In the long run, the number of individuals in class *I*_*HC*_*L*__ is far greater than that in any other class of individuals so there is need to introduce treatment so as to delay these individuals from progressing to advanced HCV and AIDS.

Our findings for the mono-infections are in agreement with those of [15]; however, for dually infected individuals, they differ. In [15], dually infected individuals start increasing and then decline asymptotically to low levels due to cure of HCV; however, for our case, individuals dually infected with HIV and latent HCV do not reduce, hence a need to introduce these individuals on treatment.

Still from Figures 11(a) and 11(b), before 50 years, there is an increase in number of individuals infected with HIV only, HCV only, and those dually infected with both HIV and HCV. This is due to increased HIV (or HCV) transmission probability per sexual contact and average number of sexual partners acquired per year. Increment in the values of these parameters leads into other parameters increasing the HIV (or HCV) infection (as earlier mentioned in Subsection 3.3.2). This could be due to individuals not knowing their HIV and HCV status. So they could acquire many sexual partners and also have unprotected sex which would increase their HIV (or HCV) transmission probability. In so doing, they would get infected and also infect others, hence an increase in the prevalence of HIV and HCV. At about 50 years, the prevalence of HIV only would be maximum whereas that of infected with HCV only and dually infected with both HIV and HCV would be maximum at about 65 years. After 50 years, the prevalence of HIV only starts reducing until it stabilises to low levels whereas that of those infected with HCV only and dually infected with both HIV and HCV start reducing until they stabilise to low levels after 65 years.

### 4.1. Observation

From simulations, HIV leveling comes after 50 years which is a long time. We are much aware that HIV dynamics have greatly changed due to the presence of HIV/AIDS treatment and in effort to achieve 90-90-90 targets. This calls for modification of the model and include treatment. This has been catered for in our currently ongoing research.

## 5. Conclusion

In this work, we formulated and analysed a mathematical model for the HIV-HCV coinfection dynamics in absence of therapy by carefully studying and analysing the HCV chronic stage split into before onset of cirrhosis and its complications and after its onset. Sensitivity analysis revealed that HIV (or HCV) transmission probability per sexual contact and average number of sexual partners acquired per year are equally likely to result into increased HIV (or HCV) infections. Furthermore, increments in the values of these parameters were the most influence among all other parameters in increasing the HIV (or HCV) infections. Therefore, for reduced HIV (or HCV) infections, individuals need to greatly reduce on the rate of sexual partner acquisition and HIV (or HCV) transmission probability per sexual contact. Numerical simulations reveal that in the long run, the number of individuals coinfected with HIV and latent HCV is far greater than that in any other class of individuals. Therefore, HIV and latently HCV-infected individuals need to seek for early treatment to slow down the progression of HIV to AIDS and latent HCV to advanced HCV. The dynamics of HIV-HCV-coinfection is dominated by HIV infection since it has a bigger basic reproduction number. This work can be extended by including treatment in the model.

## Figures and Tables

**Table 1:** HIV-HCV coinfection model parameters and their interpretations, where the appropriate units are year^−1^.

Parameter	Symbol	Value	Source
HIV transmission probability per sexual contact	*β*_1_	0.03604	[34]
HCV transmission probability per sexual contact	*β*_2_	0.05	[5]
Average number of sexual partners acquired	$\tilde{c}$	4^∗^	[24]
Rate of progression from *I*_HC_ to *I*_HC_*L*__class	*θ*	0.52	Assumed
Rate of progression from HIV to AIDS	*α*	0.07^∗^	Assumed
Rate of progression from latent to advanced HCV	∅	0.095^∗^	Assumed
Natural mortality rate	*μ*	0.0158	Calculated
Rate of progression from acute to latent HCV	*γ*	2	[18]
Recruitment rate	∧	602095	Calculated
Amplification factor for individuals in *I*_*C*_ class	*k*_1_	1.0001	[15]
Amplification factor for individuals in *I*_*H*_ class	*k*_2_	1.001	[5]
Amplification factor for individuals in *C*_*L*_ class	*k*_3_	1.0001	[15]
Enhancement factor for increased risk of being infected with HIV by a coinfected individual	*ω*	1.0002	[5]
Enhancement factor for increased risk of being infected with HCV by a coinfected individual	*ρ*	1.0002	[15]
Rate of spontaneous clearance of acute HCV	*τ*	0.27	[5]
Reduction factor for risk of acute HCV spontaneous clearance in presence of coinfection	*r*	0.25	[5]

Those with ^∗^ have $\tilde{c}\in$ [1, 4], *α*∈ [0.069, 0.1], and ∅∈ [0.095, 0.1].

**Table 2:** Sensitivity indices of  *R*_HIV_ and *R*_HCV_ with respect to parameters.

Parameter	Sensitivity index of *R*_HIV_	Parameter	Sensitivity index of *R*_HCV_
*β*_1_	+1.0001	*β*_2_	+1.0000
$\tilde{c}$	+1.0001	$\tilde{c}$	+1.0000
*α*	-0.8159	∅	-0.8123
*μ*	-0.1841	*μ*	-0.14201
		*τ*	-0.1181
		*γ*	+0.0725

## Data Availability

The parameter input values used in the simulations were obtained from literature. They are summarised in Table 1 (Subsection 3.3.1) indicating the source references.
